# Epidemiological characterization and risk assessment of rabbit haemorrhagic disease virus 2 (RHDV2/b/GI.2) in the world

**DOI:** 10.1186/s13567-024-01286-x

**Published:** 2024-03-26

**Authors:** Zhuo Sun, Qi An, Yuepeng Li, Xiang Gao, Hongbin Wang

**Affiliations:** 1 College of Veterinary Medicine, Northeast Agricultural University, Harbin, China; 2 Key Laboratory of the Provincial Education Department of Heilongjiang for Common Animal Disease Prevention and Treatment, College of Veterinary Medicine, Northeast Agricultural University, Harbin, China

**Keywords:** Rabbit haemorrhagic disease virus 2, seasonal characteristics, MaxEnt model, risk assessment

## Abstract

A novel variant of rabbit haemorrhagic disease virus, designated RHDV2/b/GI.2, was first discovered in France in 2010. Subsequently, RHDV2 rapidly spread to Africa, North America, Australia, and Asia. RHDV2 outbreaks have resulted in significant economic losses in the global rabbit industry and disrupted the balance of natural ecosystems. Our study investigated the seasonal characteristics of RHDV2 outbreaks using seasonal indices. RHDV2 is prone to causing significant outbreaks within domestic and wild rabbit populations during the spring season and is more likely to induce outbreaks within wild rabbit populations during late autumn in the Southern Hemisphere. Furthermore, based on outbreak data for domestic and wild rabbits and environmental variables, our study established two MaxEnt models to explore the relationship between RHDV2 outbreaks and the environmental factors and conducted outbreak risk predictions for RHDV2 in global domestic and wild rabbit populations. Both models demonstrated good predictive performance, with AUC values of 0.960 and 0.974, respectively. Road density, isothermality, and population density were identified as important variables in the outbreak of RHDV2 in domestic rabbits, while road density, normalized difference vegetation index, and mean annual solar radiation were considered key variables in the outbreak of RHDV2 in wild rabbits. The environmental factors associated with RHDV2 outbreaks identified in our study and the outbreak risk prediction maps generated in our study will aid in the formulation of appropriate RHDV2 control measures to reduce the risk of morbidity in domestic and wild rabbits.

## Introduction

Rabbit haemorrhagic disease (RHD) is a highly infectious, acute or subacute, rapidly fatal disease with a mortality rate of 80% to 100% [1]. It generally infects domestic and wild rabbits over the age of 2 months [2]. The disease was first observed in China in 1984 [3]. Now, it is one of the major contributors to mortality in domestic and wild European rabbits (*Oryctolagus cuniculus*) worldwide and is considered a disease that must be reported to the World Organization for Animal Health (WOAH). The disease can cause severe haemorrhages in multiple organs, pathognomonic necrosis of hepatocytes, and hyaline thrombi in renal corpuscles [4]. The etiological agent of RHD is the rabbit haemorrhagic disease virus (RHDV, genus *Lagovirus*, family *Caliciviridae*), which has a non-enveloped, single-stranded, positive-sense RNA genome.

In 2010, a new pathogenic lagovirus related to RHDV was first described in north-western France [1]. Further study of this variant indicated that it could not have evolved from RHDV, with only 85.7% average homology to RHDV [5], representing a new virus originating from an unknown pathogenic species. In 2012, a RHDV variant was identified in Spain, causing morbidity and mortality in rabbits under 30 days of age. It was suggested that the term RHDVb be used for this variant [6]. In 2013, a new pathogenic lagovirus was discovered that differed from previously described strains of RHDV and RHDVa (a variant of RHDV) in terms of phylogenetic position, antigenic profile, and pathogenicity, and it was suggested that this new lagovirus should be named RHDV2 [1]. In 2017, Le Pendu et al. established an international working group to reclassify the confusing nomenclature of lagovirus, formally categorizing RHDV2 and RHDVb as genotype GI.2 and classifying the previously described RHDV and RHDVa as genotype GI.1. Therefore, terms such as RHDV2, RHDVb, and GI.2 used in the previous references refer to the same virus [7], with the most widely accepted name being rabbit haemorrhagic disease virus 2 (RHDV2).

Since the outbreak in France in 2010, RHDV2 rapidly spread across continental Europe, such as Italy [1], Spain [6], Portugal [8], the Netherlands [9], and the United Kingdom [10], as well as to nearby islands in Europe, such as Sicily, Italy [11], Canary Islands, Spain [12], and Azores Islands, Portugal [13]. In January 2014, a strain of RHDV2 was found in dead rabbits vaccinated against RHDV in Australia, representing a transoceanic transmission of RHDV2 from Europe to Australia [14]. In February 2015, RHDV2 spread to Tunisia [15], the northernmost country in Africa, separated from Europe by the sea. In 2017, cases of RHDV2 were also reported in Morocco [16], an African country across the sea from Spain. In 2020, the epidemic continued to spread southwards in Africa, with reports from Senegal, Côte d’Ivoire, and Nigeria [17–19]. Even in 2022, South Africa [20], the southernmost country in Africa, experienced several RHDV2 outbreaks. Not only in Africa, but RHDV2 spread to North America in 2016, spreading across Canada [21], the United States [22], and Mexico [23] within 4 years (nearly 669 outbreaks), making it the most severely affected region by RHDV2 outbreaks in the world. Additionally, the occurrence of RHDV2 in Asia was reported to the WOAH by Israel [24], China [25], and Singapore [26], with no official reports of RHDV2 in Asia in the following years.

The clinical syndrome caused by RHDV2 is similar to the clinical symptoms of RHDV described in previous literature [27]. RHDV2 may have a lower mortality rate and longer incubation period than RHDV [28]. Young rabbits less than 2 months of age are more susceptible to RHDV2 than RHDV [29]. Transmission of the virus can be achieved through direct contact with infected animals, carcasses, body fluids (urine, faeces, respiratory secretions), and fur, or through mechanical vectors such as insects [30]. Disturbingly, rabbits that have been vaccinated against RHDV can still be infected with RHDV2 [31], indicating that classical RHDV immunization is only partially protective against RHDV2. Classical RHDV has a high host specificity, limited to infections in European rabbits (*Oryctolagus cuniculus*) [32]. However, RHDV2 not only infects European rabbits but has crossed the species barrier, infecting various wild rabbit species [32], such as cape hare, desert cottontail, black-tailed jackrabbit, antelope jackrabbit, mountain cottontail, mountain hare, eastern cottontail, brown hare. RHDV2 has had a considerable negative impact on ecosystems [33]. Furthermore, the recent global outbreaks of RHDV2 have resulted in substantial economic losses to the rabbit meat and fur production industries [33].

RHDV2, as an emerging transboundary disease, poses a potentially great threat to the health of wildlife and domestic animals [34]. Although it is urgent to take some preventive measures to control the spread of RHDV2, it is challenging to propose effective strategies for preventing the occurrence of RHDV2 due to the unclear epidemiological characteristics and environmental factors associated with RHDV2 outbreaks. In our study, RHDV2 outbreak data were statistically analysed, and seasonal indices were calculated to explore the epidemic seasons of RHDV2 outbreaks. We applied the MaxEnt model to assess the environmental suitability of RHDV2 by collecting data on RHDV2 outbreaks as well as bioclimatic, geographic, and socio-economic variables. The study aims to reveal the epidemiological characteristics of RHDV2 and predict the risk of future RHDV2 outbreaks, thereby assisting in the formulation of strategies for the prevention and control of RHDV2. Domestic rabbits are generally raised in fixed sites, whereas wild rabbits forage and breed freely in the wild. Considering the possible differences in the transmission and outbreak patterns of RHDV2 in domestic and wild rabbit populations, our study separately explored the seasonal prevalence of RHDV2 as well as the effects of environmental factors on outbreaks of RHDV2 in domestic and wild rabbit populations.

## Materials and methods

### Data on RHDV2

Outbreak data about RHDV2 was derived from the World Animal Health Information System (WAHIS) of the WOAH [35]. According to WAHIS, a total of 774 RHDV2 outbreaks were described globally over 13 years from 2011 to 2022. There were 611 outbreaks of RHDV2 in domestic rabbits and 163 outbreaks of RHDV2 in wild rabbits. Recorded data included event ID, administrative level (admin1, admin2, admin3, precise location), outbreak starting date, cases, deaths, rabbit species, and outbreak coordinate (longitude and latitude).

All data were collated in a database and descriptive statistics on outbreaks were completed using Microsoft Excel, version 2016. Afterward, the number of outbreaks and cases in domestic and wild rabbit populations were counted and charted by month according to the chronological order. Moreover, RHDV2 outbreaks of domestic rabbits and wild rabbits were visualized on a world map according to the latitude and longitude coordinates.

### Seasonal index (*Si*)

To investigate whether there is a specific pattern in the occurrence of RHDV2 outbreaks globally, a seasonal index (*Si*) was calculated for each month. The *Si* was calculated as the ratio between the number of RHDV2 outbreaks per month and the average number of outbreaks across all months during the study period. Outbreak data were differentiated according to the Northern and Southern Hemispheres, considering the opposite seasons. Si was calculated for RHDV2 outbreaks in the Northern and Southern Hemispheres, respectively, to explore seasonal characteristics. In addition, the *Si* of RHDV2 outbreaks in domestic rabbits, wild rabbits, and all rabbits (regardless of species) were also calculated separately.

*Si* = 1 or close to 1 indicates that RHDV2 outbreaks do not have obvious seasonal characteristics. If *Si* is much greater than 1, it means that the outbreaks in that month are often higher than the average; If *Si* is much less than 1, it indicates that the outbreaks in that month are often below the mean. In short, both cases represent a clear seasonal characteristic of RHDV2 outbreaks in that month.

### Assessing risk factors associated with RHDV2 outbreaks

#### Further cleaning of raw outbreak data

The study was conducted at a resolution of 5 arc min (approximately 10 km × 10 km), where only one record point could remain in a grid cell. Further cleaning of the latitude and longitude information of the raw outbreak data was required for successful risk modelling.

#### Processing variables for establishing risk models

To construct risk assessment models on RHDV2, 27 variables were considered, involving bioclimate, geographic, and socio-economic factors (Table 1). For bioclimatic factors, 21 bioclimatic variables were selected, including 19 bioclimate variables (bio1–bio19), mean annual wind speed (wind), and mean annual solar radiation (srad). These bioclimatic data were obtained from the WorldClim 2.1 dataset [36] with a spatial resolution of 5 arc min (approximately 10 km × 10 km). More specifically, wind and srad were obtained by calculating the average of the monthly mean wind speed and monthly mean solar radiation for 12 months using the raster calculator in the ArcGIS software. Elevation (elev), normalized difference vegetation index (ndvi), and land cover (lc) with a high resolution of 5 arc min (approximately 10 km × 10 km) were considered as geographic factors. Elevation was also accessed from the WorldClim website. Normalized difference vegetation index was provided by the Geospatial Data Cloud Platform [37]. Land cover was downloaded from the Environmental Systems Research Institute website [38]. Three socio-economic variables were considered: population density, road, and railway. Population density, sourced from LandScanGlobal dataset [39], had a high resolution of 5 arc min (approximately 10 km × 10 km). Road and railway were taken from the OpenStreetMap website [40]. Considering the transport impact on RHDV2, only major highways, second highways, beltways, and roads were extracted as the road layer. Subsequently, the road and railway layers, collected in shp format, were converted into raster layers by the kernel density analysis and resampled to 5 arc min (approximately 10 km × 10 km). The raster layers for all variables were resampled to global regions, aiming to ensure a uniform geographic scope. All operations are done in ArcGIS 10.2.

**Table 1:** **The selected and screened variables in two maximum entropy models.**

Variable category	Variable	description	Unit of measurement	Included in the model about RHDV2 in domestic rabbits	Included in the model about RHDV2 in wild rabbits
Bioclimate variables	bio1	Annual mean temperature	℃		
	bio2	Mean diurnal range	℃		
	bio3	Isothermality		Yes	
	bio4	Temperature seasonality	℃		Yes
	bio5	Maximum temperature of the warmest month	℃		
	bio6	Minimum temperature of the coldest month	℃		
	bio7	Temperature annual range	℃		
	bio8	Mean temperature of the wettest quarter	℃	Yes	
	bio9	Mean temperature of the driest quarter	℃	Yes	Yes
	bio10	Mean temperature of the warmest quarter	℃		
	bio11	Mean temperature of the coldest quarter	℃		
	bio12	Annual precipitation	mm		
	bio13	Precipitation of the wettest month	mm		
	bio14	Precipitation of the driest month	mm		
	bio15	Precipitation seasonality	%	Yes	Yes
	bio16	Precipitation of the wettest quarter	mm		
	bio17	Precipitation of the driest quarter	mm		
	bio18	Precipitation of the warmest quarter	mm	Yes	Yes
	bio19	Precipitation of the coldest quarter	mm	Yes	Yes
	wind	Mean annual wind speed	m·s^−1^	Yes	Yes
	srad	Mean annual solar radiation	kJ·m^−2^·day^−1^	Yes	Yes
Geographic variables	elev	Elevation	m	Yes	Yes
	ndvi	Normalized difference vegetation index		Yes	Yes
	lc	Land cover		Yes	Yes
socio-economic variables	popu	Population density	people·km^−2^	Yes	Yes
	road	Road density		Yes	Yes
	railway	Railway density		Yes	Yes

27 variables were selected, involving bioclimate, geographic, and socio-economic factors. After screening by the variance inflation factor (VIF), 14 environmental variables were included in the model for outbreaks of RHDV2 in domestic rabbit populations, and 13 environmental variables were included in the model for outbreaks of RHDV2 in wild rabbit populations.

The multicollinearity test was needed to avoid overfitting between variables. In this study, the variance inflation factor (VIF) of these variables was calculated using the usdm package in the R software [41]. Variables with VIF > 10 were excluded from risk models. In our study, the MaxEnt model was used to explore the risk factors for RHDV2 outbreaks. A MaxEnt model for RHDV2 outbreak in domestic rabbits and a MaxEnt model for RHDV2 outbreak in wild rabbits were developed separately.

#### Establishing MaxEnt models

The MaxEnt model is one of the most popular ecological niche models [42] and can be used to predict the distribution of pathogens and animal diseases and to identify the environmental conditions associated with disease occurrence [43]. The pattern and characteristics of RHDV2 outbreaks in domestic rabbit populations may be different from those of RHDV2 outbreaks in wild rabbit populations. Consequently, two MaxEnt models were developed, based on RHDV2 outbreak data in domestic rabbits and RHDV2 outbreak data in wild rabbits, respectively. MaxEnt 3.4.4 was employed to predict risk areas of RHDV2 outbreaks in the future as well as to identify environmental factors associated with RHDV2 outbreaks. The model was set up as follows: create response curves was ticked, make pictures of predictions was clicked, do jackknife to measure variable importance was marked, the output format was logistic, output file type was asc, the features were automatically selected, the regularisation multiplier = 1, the maximum number of background points = 10 000, the replicated run type was crossvalidate, replicates was 10, random seed was noted, and write plot data was spotted.

The prediction process of the MaxEnt model uses the maximum entropy classification algorithm, which calculates the probability of each category (outbreak or non-outbreak) based on the model parameters and the feature function. Then the category with the highest probability is selected as the prediction result. When comparing the predicted result with the actual true outbreak, the false positive (predicted outbreak but actual non-outbreak) rate is taken as the x-axis, and the true positive (predicted outbreak and actual outbreak) rate is taken as the y-axis. The curve is plotted as the Receiver Operating Characteristic (ROC) curve. The higher the rate of true positives and the lower the rate of false positives, the more accurate the MaxEnt model is. Consequently, the more the ROC curve converges to the upper left corner of the coordinate system, the more the area under the receiver operating characteristic curve (AUC) converges to 1. Therefore, the AUC is used to assess the predictive accuracy of the MaxEnt model, and it ranges from 0 to 1 [44]. Larger values of the AUC indicate higher accuracy of the MaxEnt model. The percent contribution of each variable for the model result was used to assess the importance of each variable. The variable response curves reflected the dependence of the prediction accuracy on the value range of variables [45]. The risk value of risk prediction maps for RHDV2 outbreaks ranges from 0 to 1, representing low to high risk of RHDV2 outbreaks [46].

## Results

**Figure 1:**
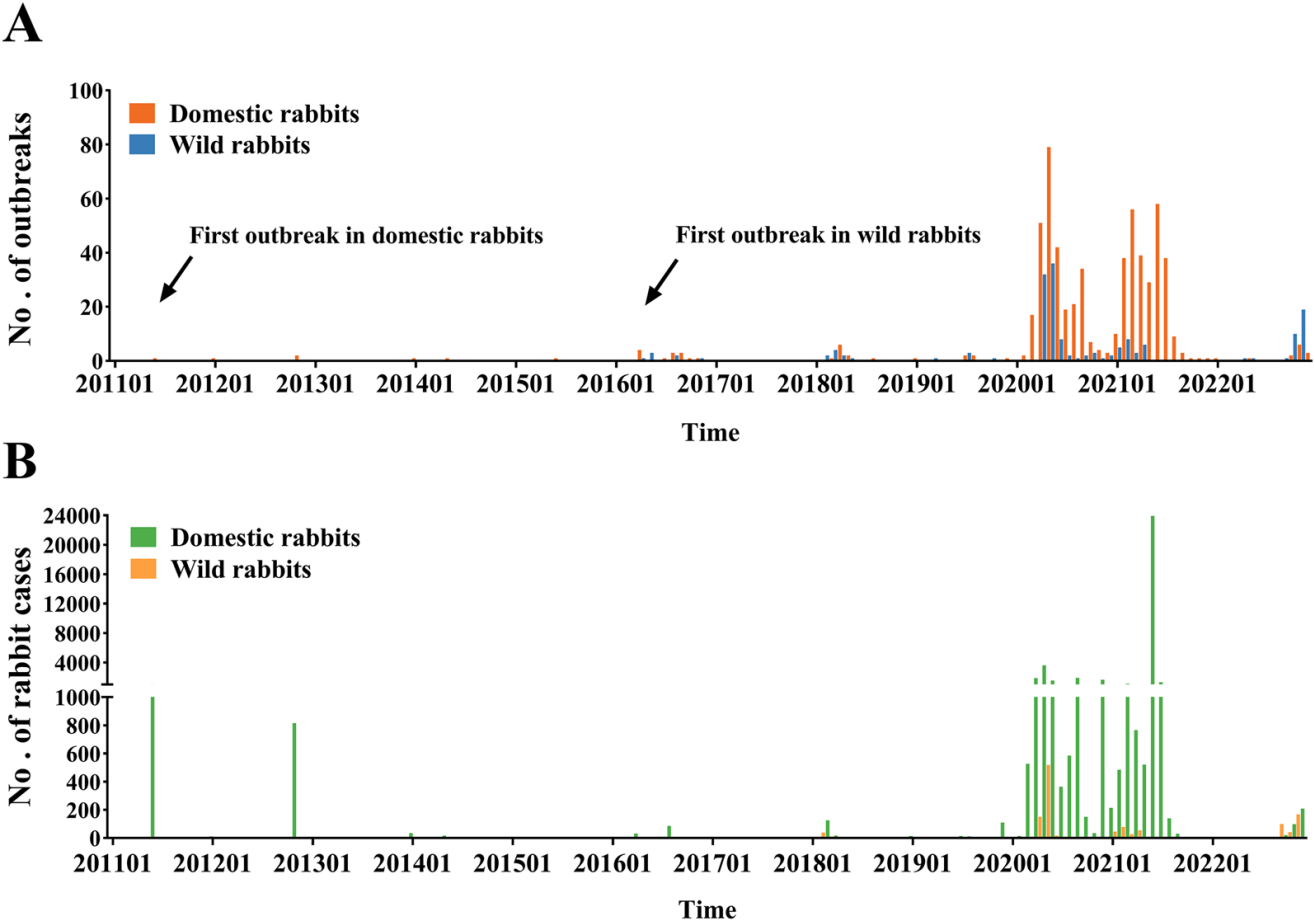
**Monthly number of rabbit haemorrhagic disease virus 2 (RHDV2) outbreaks and cases worldwide between 2011 and 2022.** The outbreak data about RHDV2 was derived from the World Animal Health Information System (WAHIS) of the World Organization for Animal Health (WOAH). **A** Outbreaks of RHDV2 in domestic and wild rabbits. The orange and blue bar charts indicate the number of outbreaks in domestic rabbits and wild rabbits, respectively. The two black arrows indicate the first outbreak in domestic rabbits and wild rabbits, respectively. **B** Cases of RHDV2 in domestic and wild rabbits. The green and yellow bar charts indicate the number of cases in domestic rabbits and wild rabbits, respectively.

**Figure 2:**
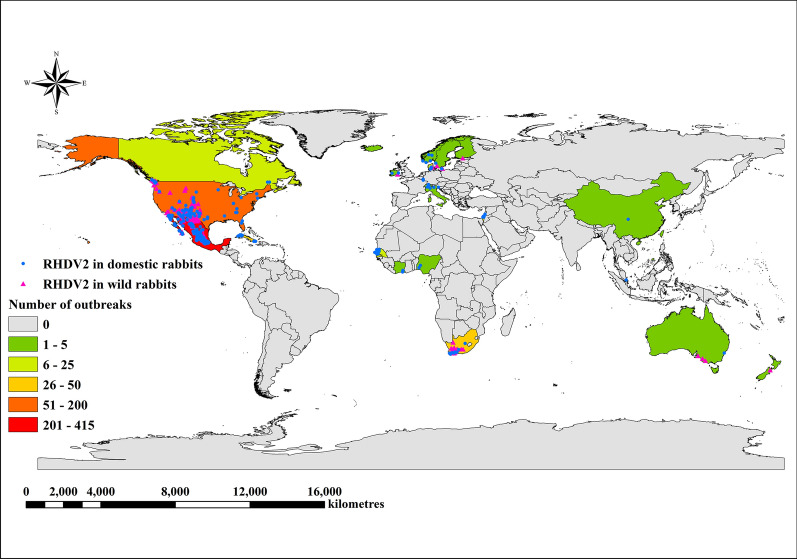
**The distribution of RHDV2 outbreaks in domestic and wild rabbits.** Blue dots represent RHDV2 outbreaks in domestic rabbits. Pink triangles represent RHDV2 outbreaks in wild rabbits. The administrative areas of countries where no RHDV2 outbreaks have occurred are shown in grey. Countries with RHDV2 outbreaks are coloured according to the number of outbreaks: dark green (1–5 cases), light green (6–25 cases), yellow (26–50 cases), orange (51–200 cases) and red (201–415 cases).

The outbreaks of RHDV2 have been sporadic worldwide from 2010 to 2019. RHDV2 outbreaks in 2020 and 2021 are frequent but non-severe compared to sporadic outbreaks in other years. 562 outbreaks of RHDV2 in domestic rabbits and 109 outbreaks of RHDV2 in wild rabbits occurred in 2020 and 2021, which accounted for 91.98% and 66.87% of the reported outbreaks in domestic rabbits and wild rabbits during the entire study period. In 2020 and 2021, a total of 40 816 domestic rabbits and 905 wild rabbits were infected with RHDV2 as reported through the WOAH, accounting for 93.72% and 69.72% of the total number of infections in domestic rabbits and wild rabbits for the entire study period, respectively. Moreover, RHDV2 outbreaks in the last quarter of 2022 were more frequent compared to the other quarters of 2022 (Figure 1). Outbreaks of RHDV2 have been reported in Europe, North America, Africa, Asia, and Australia (Figure 2). The first outbreak of RHDV2 occurred in France in 2010 and subsequently spread to other countries in Europe. Outbreaks of RHDV2 in Europe have been sporadic and non-severe. The United States and Mexico, located in North America, are the countries with the frequent but non-severe outbreaks of RHDV2. There were 198 RHDV2 outbreaks in the United States and 415 RHDV2 outbreaks in Mexico. Outbreaks of RHDV2 also occurred in Cuba in North America and South Africa in Africa, with 39 and 42 RHDV2 outbreaks, separately. In addition to the countries mentioned above, RHDV2 occurs sporadically in other countries such as Canada, Australia, Nigeria, Côte d’Ivoire, Singapore, and China.

**Figure 3:**
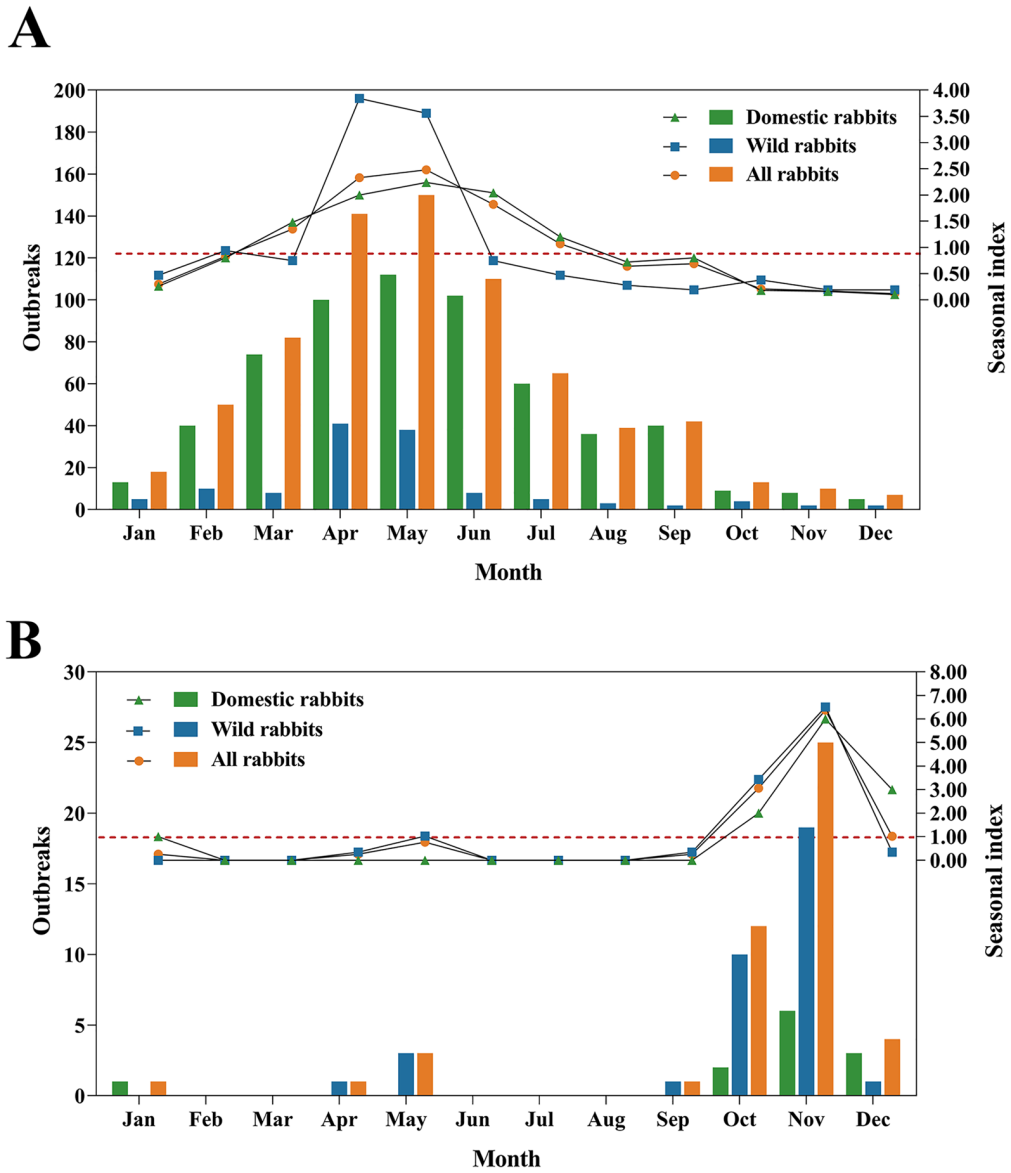
**Seasonal index (Si) of RHDV2 outbreaks in the global Northern and Southern Hemispheres.** **A** Seasonal index (*Si*) of RHDV2 outbreaks in the Northern Hemisphere. **B** Seasonal index (*Si*) of RHDV2 outbreaks in the Southern Hemisphere. The bar charts indicate the number of RHDV2 outbreaks per month. The number of outbreaks can be viewed on the left axis. Green bar charts indicate the number of domestic rabbit outbreaks per month, blue bars green bars indicate the number of wild rabbit outbreaks per month, and orange bar charts indicate the number of outbreaks in all rabbits (regardless of species) per month. The values of the *Si* are marked on the right coordinate axis. The green triangle line represents the *Si* for domestic rabbit outbreaks, the blue square line represents the *Si* for wild rabbit outbreaks, and the orange circle line represents the *Si* for all rabbit outbreaks (regardless of species). The red dotted line indicates that the value of *Si* = 1, representing no seasonal characteristics.

The seasons are reversed in the Northern and Southern Hemispheres, with spring in the Northern Hemisphere occurring from March to May and spring in the Southern Hemisphere from September to November. In the Northern Hemisphere, the highest *Si* of RHDV2 outbreaks occurred in May (*Si* = 2.24) for domestic rabbits and in April (*Si* = 3.84) for wild rabbits. In the southern Hemisphere, the highest *Si* of RHDV2 outbreaks for both domestic and wild rabbits occurred in November with *Si* of 6.00 and 6.51, respectively (Figure 3). It suggested that RHDV2 outbreaks of both domestic and wild rabbits are susceptible to springtime in both the Northern and Southern Hemispheres. Moreover, the *Si* line graph of RHDV2 outbreaks of wild rabbits in the Southern Hemisphere showed an unusually small peak in May (autumn) with *Si* = 1.03.

After further cleaning of the latitude and longitude information of the raw outbreak data, 597 outbreak points of RHDV2 in domestic rabbits and 158 outbreak points of RHDV2 in wild rabbits were included in the maximum entropy model construction. Furthermore, after filtering the environment variables by VIF, 14 environmental variables were included in the model for outbreaks of RHDV2 in domestic rabbit populations, and 13 environmental variables were included in the model for outbreaks of RHDV2 in wild rabbit populations (Table 1). MaxEnt models built in our study performed well for the prediction of RHDV2 outbreaks in domestic rabbits and wild rabbits, with AUC of 0.960 and 0.974, respectively, indicating a strong performance of the two models. The percentage contribution of the variables to the two models was employed to select important variables contributing significantly to the two models. The variables were ranked according to their percentage contribution (Table 2). Variables that accounted for more than 10 percent contribution percentage to modelling were considered significant variables. Prominent risk variables differed marginally between the two MaxEnt models based on RHDV2 outbreaks of domestic rabbits and wild rabbits. Road density (road), isothermality (bio3), and population density (popu) were considered important variables in the model constructed based on RHDV2 outbreak data in domestic rabbits, with percentage contributions of 27.8%, 19.9%, and 15.2%, respectively. Road density (road), normalized difference vegetation index (ndvi), and mean annual solar radiation (srad) were considered essential for RHDV2 outbreaks in wild rabbits, with percentage contributions of 25.4%, 17.3%, and 10.1%, respectively.

**Table 2:** **The contribution percentage of each variable to the two maximum entropy models.**

Variable	Representation	Percent contribution (%) of the variable in the MaxEnt model about domestic rabbit outbreaks	Percent contribution (%) of the variable in the MaxEnt model about wild rabbit outbreaks
bio3	Isothermality	19.9	–
bio4	Temperature seasonality	–	4
bio8	Mean temperature of the wettest quarter	0.7	–
bio9	Mean temperature of the driest quarter	1.9	6.3
bio15	Precipitation seasonality	0.7	1
bio18	Precipitation of the warmest quarter	0.3	5.9
bio19	Precipitation of the coldest quarter	5	8.3
wind	Mean annual wind speed	1.8	6.3
srad	Mean annual solar radiation	3.8	10.1
elev	Elevation	8.6	8
ndvi	Normalized difference vegetation index	6.9	17.3
lc	Land cover	1.4	1.4
popu	Population density	15.2	5.3
road	Road density	27.8	25.4
railway	Railway density	6	0.8

**Figure 4:**
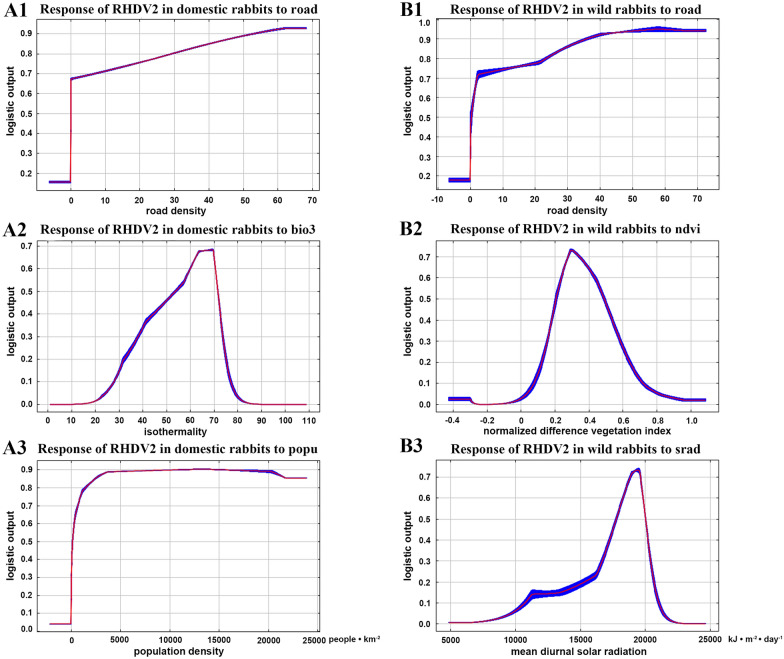
**The response curves of key variables for the two maximum entropy models.** Left plots (panels **A1**–**A3**) are the response curves of key variables related to the RHDV2 outbreak in domestic rabbits. The variables for **A1**–**A3** are road density (road), isothermality (bio3), and population density (popu). Right plots (panels **B1**–**B3**) are the response curves of key variables related to the RHDV2 outbreak in wild rabbits. The variables for **B1**–**B3** are road density (road), normalized difference vegetation index (ndvi), and mean annual solar radiation (srad).

Response curves for principal variables related to outbreaks in domestic rabbits and wild rabbits are shown in Figure 4. The horizontal axis of the response curves represents the value range of variables, and the vertical axis represents the predicted risk (range 0–1). The portion of the risk > 0.5 was considered a high predicted risk. The higher the road density, the higher the probability of RHDV2 outbreaks for both domestic and wild rabbits. Isothermality in the range of 53.70 to 71.60 had a higher possibility of RHDV2 outbreaks of domestic rabbits. In addition to this, RHDV2 outbreaks of domestic rabbits were also associated with high human population densities. Comparatively, the outbreak likelihood of RHDV2 in wild rabbits was high in places with suitable vegetation cover (0.20–0.49) and high mean annual solar radiation (17 760–20 010).

**Figure 5:**
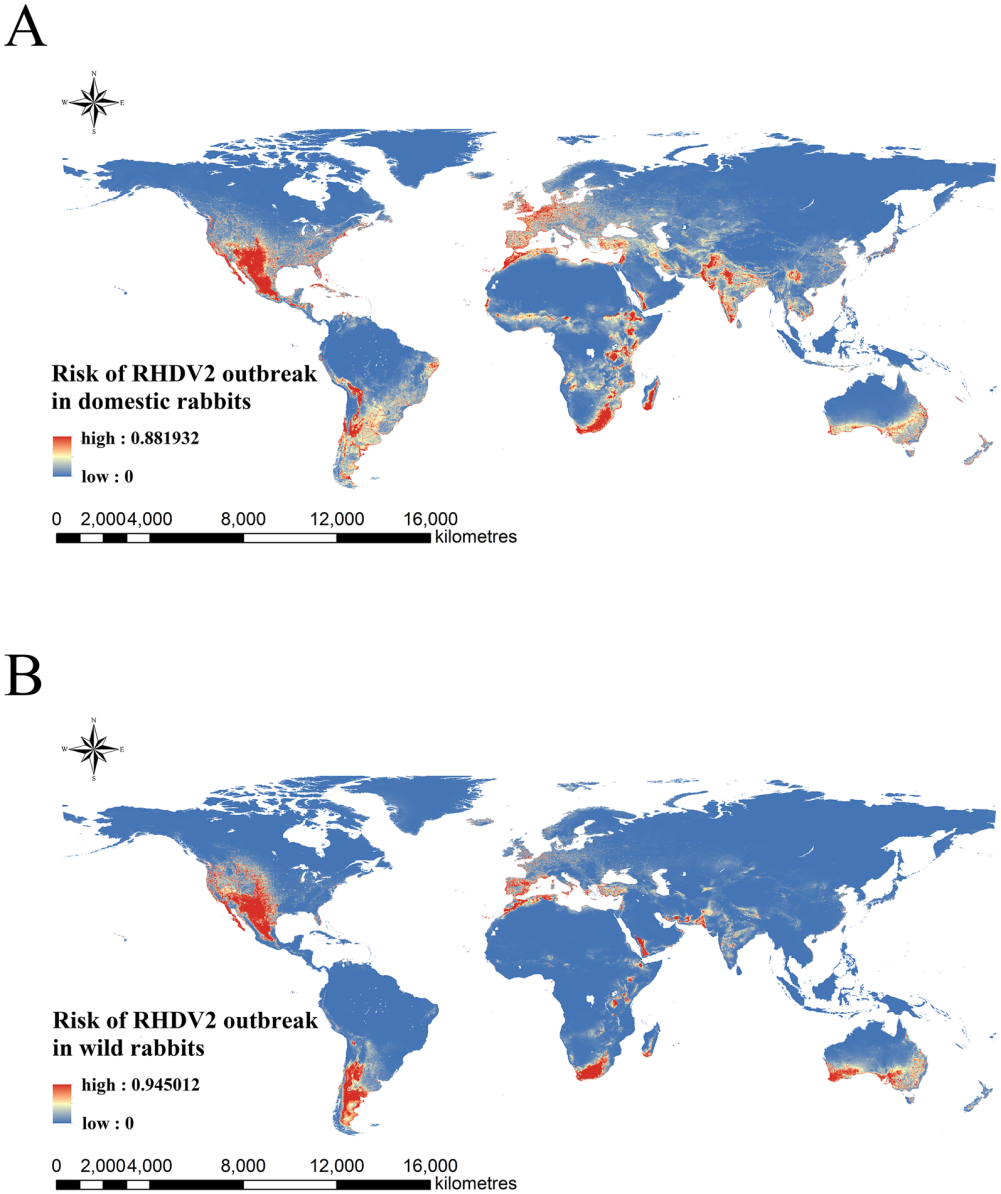
**Outbreak risk maps of RHDV2 in domestic rabbits (A) and wild rabbits (B).** The risk of outbreaks ranges from 0–1, and the risk colour transfers from blue to red on the risk maps.

The global maps of outbreak risk of RHDV2 in domestic rabbits and wild rabbits were shown in Figure 5. The results showed that the south-western regions of North and South America, the western coast of Europe, the eastern coast of Africa, the south-western coast of the Arabian Peninsula, the north-western region of the Indian Peninsula, and the southern coast of Australia were identified as high-risk areas of RHDV2 outbreaks in domestic rabbit populations. The south-west of North America, the southern region of South America, the south-west of Europe, the north-west coast of Africa across the sea from Europe, the southernmost region of Africa, the south-west coast of the Arabian Peninsula, the southern coasts of Iran and Pakistan, and the southern region of Australia were identified as high-risk areas for RHDV2 outbreaks in wild rabbit populations.

## Discussion

Since the initial discovery of RHDV2 in France in 2010 [1], RHDV2 has supplanted RHDV as the primary cause of morbidity and mortality in rabbits [47]. The WOAH has issued recommendations for the prevention and control of RHD, emphasizing that control measures primarily involve strengthening disinfection practices and implementing vaccination programs in countries where the disease cannot be eradicated. In non-endemic countries, the most critical strategy is preventing the introduction of the virus, including restrictions on the import of relevant animals and products from endemic regions [48]. Therefore, deepening our understanding of the epidemiological characteristics of RHDV2 and conducting global outbreak risk assessments for RHDV2 is of paramount importance for further controlling the spread of this disease.

So far, there has been a scarcity of research concerning the global epidemiological characteristics and risk assessment of RHDV2. Our study has illuminated the global prevalence and seasonal patterns of RHDV2, investigated environmental factors influencing outbreaks of RHDV2 in domestic and wild rabbit populations, and provided preliminary predictions of future outbreak risks on a global scale.

By examining the changing trends in the seasonal index line graph, it becomes evident that RHDV2 is prone to causing significant outbreaks within domestic rabbit populations during the spring season, both in the Northern and Southern Hemispheres. Domestic rabbits do not exhibit seasonal oestrus, as they can breed throughout the year. In contrast, spring offers favourable climatic conditions characterized by mild temperatures and ample sunlight, making it the optimal breeding season for domestic rabbits. It is highly susceptible to triggering extensive outbreaks of RHDV2 within juvenile rabbit populations during the spring season, as RHDV2 readily infects rabbits aged below 2 months [2]. Seasonal indices of RHDV2 outbreaks in wild rabbit populations in both the Northern and Southern Hemispheres also display clear peaks in spring. It suggests that RHDV2 also tends to outbreak in wild rabbit populations during spring in both the Northern and Southern Hemispheres. The pattern can be attributed to the natural reproductive cycles of wild rabbits, which typically give birth offspring during spring due to dry conditions and favourable temperatures, as well as increased availability of natural forage [49]. The amount of green forage will be more abundant in the summer than in the spring, providing more protein for wild rabbits. However, RHDV2 outbreaks did not become prevalent in the summer. The reason may be that the higher temperatures and increased precipitation in summer affect the transmission of RHDV2. Unexpectedly, RHDV2 outbreaks in wild rabbit populations in the Southern Hemisphere also show a small peak in May. The RHDV2 outbreak data of wild rabbits for the Southern Hemisphere in our study came from three main countries, South Africa, Australia and New Zealand. The RHDV2 outbreaks of wild rabbits in South Africa all occurred in October and November (spring). The small peak of RHDV2 outbreaks in the Southern Hemisphere in May was derived from outbreak data in Australia and New Zealand. It indicates that RHDV2 may also be an epidemic in wild rabbits during autumn in Australia and New Zealand. Indeed, we consider that actual RHDV2 outbreaks of wild rabbits in Australia should be underestimated, due to the limited number of RHDV2 outbreaks of wild rabbits in the Southern Hemisphere obtained from the WOAH website. The predicted probability of IgM seropositivity detected in RHDV2 dominant environments of Australia peaks in autumn, revealing that RHDV2 tends to start outbreaks in autumn, resulting in the emergence of short-lived antibody IgM in young rabbits [29].

Our research results indicate that the RHDV2 outbreaks within domestic rabbits are associated with proximity to roads, higher human population density, and environments characterized by suitable isothermality. The positive correlation between RHDV2 outbreaks in domestic rabbits and road density and population density intuitively reflects the promoting role of human activities in the occurrence and spread of this disease. Regions with high human population density typically exhibit increased demand for rabbit meat and products, leading to larger rabbit farming operations and an elevated likelihood of RHDV2 infection. Previous research has demonstrated strong environmental adaptation of RHDV2, as it can survive for extended periods, even in freezing conditions, such as in decaying carcasses or frozen meat, for several months [30]. Consequently, the transportation and export of rabbit meat and products may contribute to the virus's spread into new geographic areas [30]. During transportation, the virus may contaminate the environment around roads, resulting in infections and deaths among domestic or wild rabbits in the environment in proximity to roads. The rapid global spread of this virus within a few years is highly likely to be linked to the transportation of live rabbits and their products or human population migration [50]. Isothermality is defined as one hundred times the ratio of the daily temperature range to the annual temperature range. Changes in isothermality can explain the warmth of the climate. The probability of RHDV2 outbreaks in domestic rabbits is higher when isothermality falls within the range of 53.70 to 71.60. It suggests a close relationship between warm climates in regions of moderate to low latitudes and the occurrence of RHDV2 outbreaks. In regions of moderate to low latitudes, such as Europe and Asia, the rabbit breeding industry is well developed. With such a large and susceptible rabbit population, the outbreak probability of RHDV2 is even larger. It is unlikely to be due to the virus itself. UV light is very strong at low and middle latitudes, which is not conducive to virus survival.

The outbreak of RHDV2 in wild rabbit populations also maintains a positive correlation with road density, which may be related to rabbit habitat habits and human activities. Actually, in intensively farmed/grazed or urbanized areas, road verges are the only remaining habitat for small mammals like wild rabbits [51]. The rich vegetation and shrubs along roadsides provide food and shelter for wild rabbits. Meanwhile, the transportation of contaminated live rabbits and their products may increase the infection risk of wild rabbits in areas around roads. Alternatively, there may be a higher likelihood of discovering dead wild rabbits in proximity to roads. Further exploration of the reasons behind the association between RHDV2 outbreaks in wild rabbit populations and roads will be necessary in subsequent research. RHDV2 outbreaks in wild rabbit populations are also associated with suitable vegetation coverage (0.20–0.49) and higher mean annual solar radiation (17 760–20 010). It is likely related to the habitat preferences and behaviour of wild rabbits. Wild rabbits prefer habitats such as mixed woodlands with water sources, areas with concealment in shrubbery, grassland regions, and slopes near farmlands and vegetable fields [52, 53]. Vegetation coverage in these areas tends to be moderate [54], allowing them to watch for danger and escape rapidly. Additionally, wild rabbits favour dry environments and ample sunlight, which can enhance their metabolism and reduce the occurrence of many diseases [55]. Regions at low to moderate latitudes with frequent sunny days and low humidity typically experience higher mean annual solar radiation, promoting the growth of wild rabbits and increasing the likelihood of RHDV2 infection.

Currently, regions with well-developed rabbit industries are primarily found in Europe and Asia [56]. Europe, as the traditional main producer of meat rabbits worldwide, has consistently been heavily affected by RHDV2 outbreaks. The ongoing local sharing of RHDV2 strains between domestic and wild rabbits in Europe may potentially drive the evolution of the virus, increasing the average virulence of RHDV2 [57]. In Europe, both Spain and France have licensed highly effective inactivated vaccines to combat RHDV2 [58]. Regular and organized vaccination efforts against RHDV2 are crucial for controlling the spread and evolution of RHDV2 in Europe. In contrast, official reports of RHDV2 cases in Asia have been relatively scarce. However, since 1990, the rabbit industry in Asia has entered a phase of rapid expansion, making it the continent with the largest rabbit production globally [59]. China contributes significantly to the rabbit farming industry in Asia, accounting for 80% of the total rabbit production in the Asian region [59]. In China and other regions of Asia, it is necessity to elevate the awareness of RHDV2 and actively engage in regular serological monitoring as well as monitoring RHDV2 prevalence and evolution within wild rabbit populations.

RHDVa was introduced as the biological control agent in the early stages in Australia to control the population of wild rabbits [60]. In Australia, domestic rabbits are vaccinated against RHDVa to prevent infection and mortality caused by RHDVa. Over the eight years since the spread of RHDV2 to Australia, it has played a significant role in suppressing wild rabbit populations. However, it is not feasible for RHDV2 to continue exerting a greater impact on the control of wild rabbit populations, as it is likely that rabbit populations will eventually recover [61]. Furthermore, the inactivated RHDVa vaccines do not provide cross-protection against RHDV2 [62], which means that rabbits vaccinated with RHDVa are still at risk of infection with RHDV2. There is currently no widely used local RHDV2 vaccines in Australia, apart from a very small number of imported RHDV2 vaccines. Overall, the role of RHDV2 in controlling wild rabbits has been diminished, but the threat to Australian domestic rabbits has increased. In an immunological cross-protection trial of different types of RHDV conducted in Australia, it was found that the inactivated vaccine lacked cross-protection, whereas the multivalent vaccine demonstrated good cross-protection [63]. Therefore, in addition to strengthening virus monitoring and preventive measures, it is highly urgent for the development, evaluation, and licensing of a multivalent RHDV vaccine that protects rabbits from multiple genotypes of RHDV.

In addition to several endemic areas with high outbreak risk, the southern part of South America also has a high outbreak risk through examining the outbreak risk prediction maps. While there are currently no official reports of RHDV2 outbreaks in South America, the region has a higher rabbit farming population compared to North America, characterized by extensive farming practices and lower technological standards [64]. It is advisable to strengthen the monitoring of RHDV2 in South America and formulate emergency preparedness plans.

Our study also has some limitations. All outbreak data for RHDV2 were obtained from the WOAH. During the early stages of RHDV2 outbreaks in Europe, there may have been underreporting due to a lack of precise knowledge about RHDV2. It may lead to sampling bias and affect the results of the MaxEnt model. Some research suggests that insects may potentially serve as mechanical vectors in short-distance RHDV2 transmission [65]. If the role played by insects in RHDV2 outbreaks needs to be explored in this experiment, global insect distribution points need to be collected, which is very difficult for us. Moreover, it is necessary to identify which specific insects are likely to mechanically transmit RHDV2 before collecting sites, and the literature on insects that can mechanically transmit RHDV2 is not yet extensive. Therefore, only the effects of global climatic, geographic and socio-economic factors on RDHV2 outbreaks were explored, and not the effects of vectors on RHDV2 outbreaks. Our study is indeed deficient in this aspect. The relationship between RHDV2 outbreaks and insects in the United States and Mexico will be explored in subsequent studies. It is feasible to collect insect distribution data in the United States and Mexico. After collecting insect survival sites in the United States, it will be possible to predict insect suitability zones in the United States and Mexico. By using the insect niche in the United States and Mexico as one of the independent variables in the outbreak risk assessment model, it will be possible to determine whether RHDV2 outbreaks are associated with which specific insect species. In the future, our study will consider the effect of insects for transmission vectors and the distribution density of domestic rabbits on RHDV2 outbreaks to more accurately predict RHDV2 outbreak risk.

RHDV2 is prone to causing significant outbreaks within domestic and wild rabbit populations during the spring season and is more likely to induce outbreaks within wild rabbit populations during late autumn in the Southern Hemisphere. Road density, isothermality, and population density are considered important variables in the outbreak of RHDV2 in domestic rabbits. Road density, normalized difference vegetation index, and mean annual solar radiation are regarded as key variables in the outbreak of RHDV2 in wild rabbits. This study contributes to the targeted prevention and control efforts in areas at high risk of RHDV2 outbreaks.

## Data Availability

The datasets generated and/or analyzed during the current study are available from the corresponding author upon reasonable request.
